# 0995. Aspirin reduces neutrophilic pulmonary inflammation in a human model of acute respiratory distress syndrome induced by inhaled lipopolysaccharide

**DOI:** 10.1186/2197-425X-2-S1-P80

**Published:** 2014-09-26

**Authors:** U Imran Hamid, J Conlon, S Spence, A Krasnodembskaya, A Kissenpfennig, DF McAuley, CM O'Kane

**Affiliations:** Centre for Infection and Immunity, School of Medicine, Dentistry and Biomedical Sciences, Queen's University of Belfast, Belfast, UK

## Introduction

Acute respiratory distress syndrome (ARDS) is characterized by damage to the alveolar epithelial-endothelial barrier resulting in neutrophil influx and pulmonary oedema. The activation of platelet and secondary capture of neutrophils may play an important role in propagation of inflammation in ARDS [1]. Various animal studies have shown that aspirin therapy reduces pulmonary oedema and development of lung injury [2]. In observational studies, patients on aspirin therapy prior to hospital admission had a reduced incidence of ARDS [3]. By acetylating cyclooxygenase, aspirin inhibits platelet aggregation and generates anti-inflammatory molecules which modulate neutrophilic inflammation [4].

## Objective

To test the hypothesis that aspirin reduces pulmonary inflammation in an *in vivo* human model of acute respiratory distress syndrome, induced by inhaled lipopolysaccharide (LPS).

## Methods

Healthy subjects were enrolled in a double-blind, placebo-controlled study and were randomised to receive aspirin 75mg or aspirin 1200mg or placebo (1:1:1) for seven days prior to LPS inhalation. Measurements were performed in bronchoalveolar lavage (BAL) fluid obtained at 6 hours after inhaling 50 micrograms of LPS.

## Results

**Figure 1:**
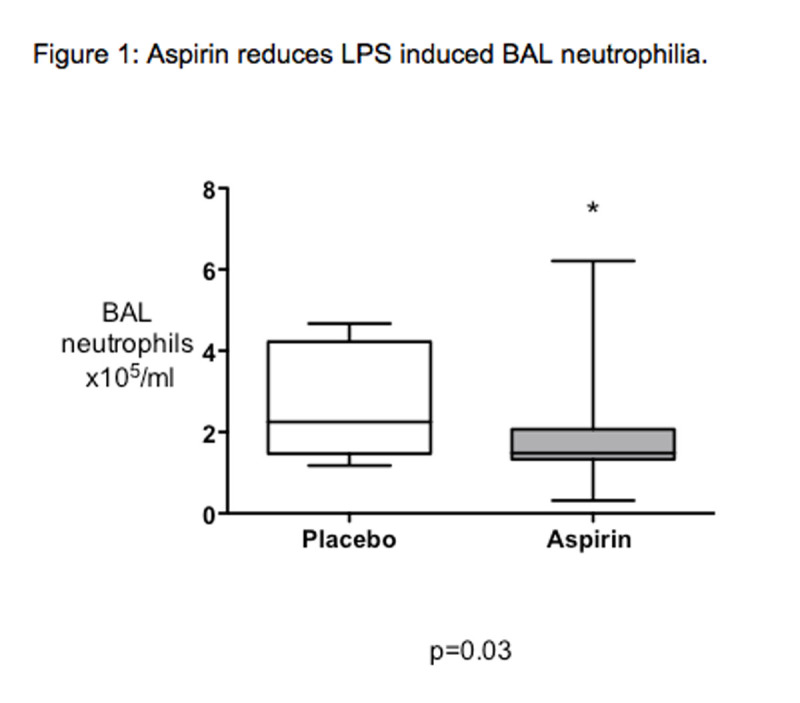
Aspirin reduces LPS induced BAL neurtophilia.

33 healthy subjects were enrolled. There was no significant difference between aspirin 75mg and aspirin 1200mg. Data for both aspirin groups were combined. Aspirin pre-treatment reduced LPS induced BAL neutrophilia (figure 1) and BAL concentrations of both the neutrophil-specific protease MMP-8, and the pro-inflammatory cytokine TNF-α. There was a non-significant trend towards reduction in a range of inflammatory cytokines (table 1).

**Table 1:** Effect of aspirin on markes of pulmonary inflammation

BAL				
	Placebo (n=13)	Aspirin (n=20)	% reduction	p-value
PMN (10^5^cells/ml)	2.25 (1.18, 4.67)	1.48 (0.32, 6.21)	34.2	0.03
TNF-α (pg/ml)	105.7 (73.49, 358.5)	79.72 (37.34, 276.6)	24.5	0.02
IL-6 (pg/ml)	856.5 (535.9, 2285)	647.9 (224.6, 1582)	24.1	0.07
MMP-8 (pg/ml)	6342 (1932, 9574)	2902 (862.8, 6758)	54	0.03
MMP-9 (pg/ml)	48493 (23126, 99780)	33717 (10471, 72334)	30.4	0.03
IL-8 (pg/ml)	447.5 (236.9, 1250)	345.9 (175.5, 1522)	22.7	0.11
IL-1β (pg/ml)	42.74 (25.98, 98.76)	37.03 (18.73, 54.88)	13	0.16

## Conclusion

This study shows for the first time that aspirin can reduce neutrophilic inflammation in the lung in humans. Further clinical studies are warranted to assess its ability to reduce neutrophil mediated inflammation in ARDS.
